# An additional tilted‐scan‐based CT metal‐artifact‐reduction method for radiation therapy planning

**DOI:** 10.1002/acm2.12523

**Published:** 2018-12-31

**Authors:** Changhwan Kim, Rizza Pua, Chung‐Hwan Lee, Da‐in Choi, Byungchul Cho, Sang‐wook Lee, Seungryong Cho, Jungwon Kwak

**Affiliations:** ^1^ Department of Nuclear and Quantum Engineering KAIST Daejeon Republic of Korea; ^2^ Department of Radiation Oncology Asan Medical Center Seoul Republic of Korea; ^3^ Department of Radiation Oncology Asan Medical Center University of Ulsan College of Medicine Seoul Republic of Korea

**Keywords:** CT, metal artifact reduction, radiotherapy

## Abstract

**Purpose:**

As computed tomography (CT) imaging is the most commonly used modality for treatment planning in radiation therapy, metal artifacts in the planning CT images may complicate the target delineation and reduce the dose calculation accuracy. Although current CT scanners do provide certain correction steps, it is a common understanding that there is not a universal solution yet to the metal artifact reduction (MAR) in general. Particularly noting the importance of MAR for radiation treatment planning, we propose a novel MAR method in this work that recruits an additional tilted CT scan and synthesizes nearly metal‐artifact‐free CT images.

**Methods:**

The proposed method is based on the facts that the most pronounced metal artifacts in CT images show up along the x‐ray beam direction traversing multiple metallic objects and that a tilted CT scan can provide complementary information free of such metal artifacts in the earlier scan. Although the tilted CT scan would contain its own metal artifacts in the images, the artifacts may manifest in a different fashion leaving a chance to concatenate the two CT images with the metal artifacts much suppressed. We developed an image processing technique that uses the structural similarity (SSIM) for suppressing the metal artifacts. On top of the additional scan, we proposed to use an existing MAR method for each scan if necessary to further suppress the metal artifacts.

**Results:**

The proposed method was validated by a simulation study using the pelvic region of an XCAT numerical phantom and also by an experimental study using the head part of the Rando phantom. The proposed method was found to effectively reduce the metal artifacts. Quantitative analyses revealed that the proposed method reduced the mean absolute percentages of the error by up to 86% and 89% in the simulation and experimental studies, respectively.

**Conclusions:**

It was confirmed that the proposed method, using complementary information acquired from an additional tilted CT scan, can provide nearly metal‐artifact‐free images for the treatment planning.

## 1 INTRODUCTION

Metal artifacts in x‐ray computed tomography (CT) scans are due to the presence of high‐density objects, such as dental fillings, hip prostheses, and surgical clips, within the scanning field of view (FOV).^1^, ^2^, ^3^ These artifacts are caused by multiple physical factors, including photon starvation, scatter, beam hardening, and noise. Metal artifacts in the reconstructed CT images are typically observed as streaks and bright/dark band artifacts near metallic objects. The presence of metal artifacts in CT images adversely affects image quality, with the appearance of low‐contrast structures near the metallic implants being especially deteriorated.^2^ Reduced image quality prevents accurate delineation of structures, markedly reducing the diagnostic value of CT scans and the accuracy of dose calculations for radiation treatment planning.

Occasionally, unavoidable circumstances, such as the inclusion of metal implants in the treatment field, add up to the challenges of radiation therapy in delivering accurate dose to the tumor target while sparing the surrounding tissues. The detrimental effects of metal artifact‐corrupted CT images have already been demonstrated in radiation therapy (RT) by numerous studies.^4^, ^5^, ^6^, ^7^, ^8^, ^9^, ^10^, ^11^, ^12^, ^13^, ^14^, ^15^, ^16^, ^17^, ^18^, ^19^, ^20^ Furthermore, the performance of various metal artifact reduction (MAR) techniques applied in these studies have been evaluated with respect to accurate tissue delineation and dose calculation. Patients undergoing pelvic irradiation for prostate cancer treatment, for instance, may have a unilateral or bilateral prostheses. Using uncorrected CT images, Su et al. demonstrated that intensity modulated radiation therapy (IMRT) treatment plans were superior to the conventional three‐dimensional conformal radiation therapy (3D‐CRT) plans in delivering the planned dose and sparing the organs at risk (OARs) in an early stage prostate cancer patient with bilateral hip implants.^4^ The IMRT plans involved a tedious task of preventing the beams from passing through the metals. However, IMRT plans still delivered more than 105% of the prescribed dose to the target volume. A homogenous pelvic phantom‐based study by Ding and Yu showed an underestimation of CT numbers of metal implants.^5^ Without any corrections to the CT image, an overestimated dose was calculated by a commercial analytical 3D treatment planning system (CADPLAN) and was delivered to the target volume. Inaccuracy of CT numbers is one of the common problems encountered by medical physicists in constructing successful treatment plans for patients. For instance, dosimetric errors from 6 and 18 MV radiotherapy (RT) plans in the target volume of prostate patients with bilateral hip prostheses were reported by Wei et al.^6^ The target volume for both photon beam energies was undermined due to the metal artifact‐corrupted CT images used in the treatment planning. Also, 6 MV four‐field RT plans were shown to be more susceptible to the metal artifacts than 18 MV four‐field RT plans. Likewise, OARs are considered as major concerns in RT planning. Without metal artifact suppression, the target volume and OARs receive underestimated dose and overestimated dose, respectively. These dose perturbations were also manifested in the Monte Carlo (MC) dose calculations of bilateral prostheses phantom studies and prostate patient study at the same photon energies conducted by Bazalova et al.^7^ After implementing a sinogram inpainting‐based MAR, identification and delineation of the target tumor and OARs became more straightforward than utilizing the artifact‐contaminated CT images. With an additional extended calibration to aid and increase the MC dose calculation accuracy, the dosimetric error in 6 MV RT plan dropped from 25% in uncorrected CT images to about 2% in MAR‐corrected CT images. The improvement in dose calculations was also seen in the 18 MV case. These dose perturbation issues arising from the pelvic irradiation of patients with hip prostheses were also addressed in Task Group 63 of Association of Physicists in Medicine (AAPM) Radiation Therapy Committee (RTC).^8^

Generally in clusters and small in size, highly attenuating dental filling materials (DFMs) considerably influence the CT images of oral cavity and head‐and‐neck (H&N) regions. Calculated dose from CT images of these regions resulted in significant dose increase to the OARs due to backscatter from the DFMs and decrease in the target tumor coverage due to the high attenuation property of DFMs. These anomalies were reduced after applying a mask that forces the metal streak artifacts to a soft tissue value of 10 HU, and applying a virtual filter that compensates for the beam attenuation of DFMs. Specifically, the mask improved the dose homogeneity while the virtual filter enhanced the delivered dose to the target tumor. These findings were obtained from the phantom and patient studies using RapidArc RT plans in the successive studies by Mail et al.^9^, ^10^ In another dental phantom study by Maerz et al., dose distribution deviations were calculated in both IMRT and volumetric modulated arc therapy (VMAT) plans generated from metal artifact‐contaminated CT images.^11^ Their study also concluded that RT plans created from metal artifact‐corrected CT images resulted in a significant decrease in dose perturbations for the H&N cases. In terms of accuracy of dose calculations, VMAT exhibited a closer dose distribution agreement with the reference film measurement data than IMRT.

Spine implants are low‐ or high‐Z metals with a complex geometry, usually situated within or near the target volume. Therefore, delineation of both target volume and OARs has always been a difficult task due to the metal artifacts. Son et al. indicated that an average of 2% dose calculation discrepancy between the implants and increasing dose errors toward the location of an implant were observed in their phantom study.^12^ A clinical study conducted by Spadea et al. revealed that low‐ and high‐Z metal implants affect dose perturbations differently in uncorrected CT images.^13^ An MAR approach, incorporating the metal material information, developed by Verburg and Seco was implemented to reduce metal artifacts in the patient CT images.^14^ For low‐Z metal spine implants, no significant dose discrepancy was exhibited between IMRT plans created from artifact‐contaminated and artifact‐corrected CT images. For high‐Z metal implant, however, that is, gold dental fillings and platinum wire for artery embolization, dose errors as high as 20–25% were calculated near the implants.

Compared to the x‐ray external beam radiation therapy (EBRT) and brachytherapy, treatment plans for proton therapy and heavy ion therapy substantially rely on accurate stopping power derived from CT numbers of materials along the beam path to calculate the dose distribution and beam ranges. However, CT number accuracy decreases with the presence of metal artifacts inducing errors in the target coverage and lessening the sparing of normal tissues. Phantom studies by Jakel and Reiss exhibited that the metal artifacts alone generated by dental fillings, titanium hip implants, and steel hip implants underestimated the ion beam range by as much as 3%, up to 5% and 18%, respectively.^15^ Verburg and Seco reported that errors in the beam range caused by titanium spine implants were also dictated by the geometry of the implant and proton beam orientation relative to the implant and artifacts.^16^ In their phantom study, proton beams traversing through metal implants and bright streak artifacts or parallel to the artifacts resulted in large range errors from 1 to 10 mm. On the other hand, beams perpendicular to metal artifacts caused insignificant effect on the dose calculations. Clinical dose calculations of their patient study showed range errors up to 6 mm in regions distant from the artifacts. Similar findings regarding minimal range errors were reported by Lin et al. when the proton beam is oriented perpendicular to or at 60‐degree angle with respect to the Ti‐mesh cranial implants.^17^ Range errors of 5–12 mm were also calculated by Newhauser et al. in prostate treatment plans of patients with unilateral or bilateral hip prostheses after utilizing metal artifact‐corrupted kVCT image.^18^ A hybrid kVCT–MVCT‐based treatment plan was recommended for easier tissue delineation and smaller ion range error. In a patient study with bilateral hip prostheses, Wei et al. showed that an under‐dosed target volume between the implants was brought by using metal‐contaminated CT image for treatment planning.^6^ Applying a previously proposed MAR (Wei et al.), a variation of 13 and 9 mm, respectively, from the uncorrected datasets were calculated for the beam range and modulation.^19^ A compilation of different studies from EBRT, brachytherapy, proton, and heavy ion RT for different metal implants and the benefits of MAR for such investigations are well summarized in the work of Giantsoudi et al.^20^

Among the MAR methods recently investigated are sinogram inpainting,^1^, ^19^, ^21^, ^22^, ^23^, ^24^, ^25^, ^26^, ^27^, ^28^, ^29^, ^30^ iterative,^31^, ^32^, ^33^, ^34^, ^35^, ^36^, ^37^ and hybrid^2^, ^3^, ^38^, ^39^, ^40^, ^41^, ^42^, ^43^, ^44^ methods. Sinogram inpainting methods are the most common MAR algorithms, in which sinogram data containing the metal traces are replaced by using interpolation or forward projections. Iterative methods use different reconstruction models to solve ill‐posed problems with a relevant regularization, allowing iterative reconstruction of images from uncorrupted projections alone. Hybrid methods, combining the advantages of existing methods, have also been utilized. Current MAR methods, however, have not attained broad clinical use, because none is able to completely remove metal artifacts in every situation.^41^ Although existing methods may remove metal artifacts in some cases, they may introduce new artifacts or false structures, or even degrade image quality, in other cases.

This study proposes a novel approach, which utilizes data from an additional tilted CT scan, to MAR. This new method is based on the fact that most metal artifacts in CT images are caused by the object's high attenuation on traversing beams in CT scans. Therefore, tilted CT scans would provide information complementary to that of scans in which some regions are free of metal artifacts. Using the two images, a combined artifact‐free image with much reduced metal artifacts can be generated. This study utilized a modified version of structural similarity (SSIM) as an index to select the regions with less metal artifacts. Quantitative analyses in both simulations and experiments were conducted to show that the proposed method effectively reduces metal artifacts in the reconstructed images.

## 2 MATERIALS AND METHODS

### 2.A Idea

The main idea underlying the proposed method is that tilted CT scans can provide complementary image data free of metal artifacts in the regions that have been contaminated in the original CT image. Metal artifacts appear different in the reconstructed images obtained from CT scans at varying system‐tilt angles.^45^ These differences are due to the effects of physical factors that cause metal artifacts, including photon starvation, beam hardening, scatter, and noise, all of which are subject to change as the scanner tilt angle is altered. Therefore, tilted CT scans can provide information complementary to that of the standard CT scans, with the tilted CT images being free of metal artifacts and the standard CT images containing the metal artifacts. By selecting regions with less metal artifacts between the two images, images nearly free of artifacts can be generated by combining the two CT images.

A reconstructed image at an ordinary 0‐degree gantry tilt‐angle contains the metal artifacts of an ordinary scan, whereas an image acquired at a tilted angle may be composed of the artifact‐free image in the contaminated regions in an ordinary scan and the metal artifacts from an oblique scan. Because the object structures in the two images would be nearly identical, differences between the two images would be due only to the metal artifacts. Therefore, difference between the two reconstructed images would constitute a superposition map of metal artifacts from a standard CT and an oblique CT scan. This superposition map would have no structural information about the scanned object, but would only contain the superposition of metal artifacts from the two images.^46^ The correlation maps from each reconstructed image and superposition map would describe the degree of contained artifacts in the relevant reconstructed image. That is, a higher value in the correlation map would indicate that the corresponding reconstructed image contains more artifacts. Therefore, the regions chosen from the two correlation maps with lower correlation values would be a template for artifact‐free image.

A modified version of SSIM was used as an index to calculate the degree of correlation between the reconstructed images and their corresponding artifact superposition maps.^47^ SSIM was designed to calculate the similarity between two images by measuring three types of visual perception: luminance, contrast, and structure.

(1) $$SSIM\left(x,y\right)=l{\left(x,y\right)}^{\alpha }c{\left(x,y\right)}^{\beta }s{\left(x,y\right)}^{\gamma },$$

where $l{\left(x,y\right)}^{\alpha }$, $c{\left(x,y\right)}^{\beta }$, and $s{\left(x,y\right)}^{\gamma }$ are luminance, contrast, and structure factors, respectively. The individual factors can be calculated as:

(2) $$l\left(x,y\right)=\frac{2\mu_{x}\mu_{y}+c_{1}}{\mu_{x}^{2}+\mu_{x}^{2}+c_{1}},$$

(3) $$c\left(x,y\right)=\frac{2\sigma_{x}\sigma_{y}+c_{2}}{\sigma_{x}^{2}+\sigma_{x}^{2}+c_{2}},$$

(4) $$s\left(x,y\right)=\frac{\sigma_{\mathrm{xy}}+c_{3}}{\sigma_{x}\sigma_{y}+c_{1}},$$

where $\mu_{y}$, $\sigma^{2}$, and $\sigma_{\mathrm{xy}}$ are the average, variance, and covariance, respectively. The luminance factor is associated with the average value or intensity of each image; the contrast factor is associated with the variance of each image; and the structure factor is associated with the covariance of two images. However, the original SSIM cannot be directly applied, because the intensity values of the areas containing the metal artifact in the CT images and the superposition map differ significantly, whereas their structural or edge information is relatively identical. Therefore, the correlation value calculated by the original SSIM may not accurately represent the degree of metal artifacts included in the corresponding image (see Appendix). Thus, this study excluded the luminance factor in the original SSIM; rather, it utilized a modified SSIM containing contrast and structure factors to calculate the correlations.

The proposed method would be applicable to the situations of sufficient complementary information. In most cases, an additional tilted CT scan would provide adequate regions for MAR. However, in some cases, it may be difficult to obtain sufficient complementary information with additional tilted CT scans alone, such as when severe metal artifacts occur in both standard and tilted CT images. For example, if the direction or magnitude of the tilt angle is limited, or if the metal artifacts are too severe, the tilted CT image would still contain metal artifacts. As the proposed method would show imperfect performance of MAR in such cases, incorporation of this method into an existing MAR method may provide a solution. The sinogram inpainting method is a potential candidate, because its results provide reasonably compliant information to replace the regions of residual metal artifacts, despite the results of the sinogram inpainting method not being completely artifact‐free. Additional details are provided in Sections Results and Conclusions.

### 2.B Algorithm

**Figure 1:**
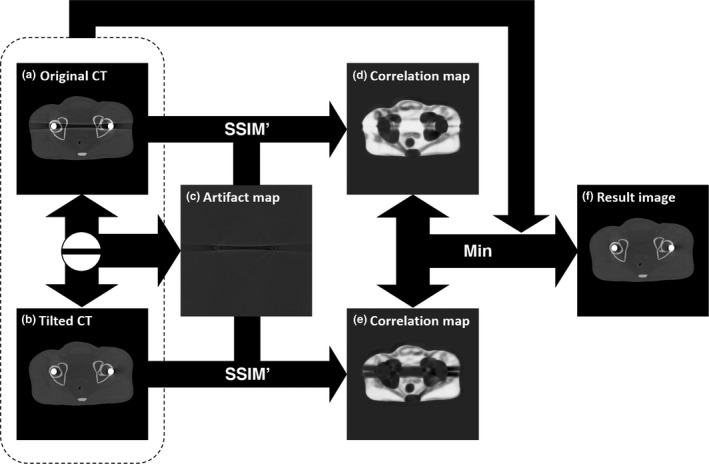
Conceptual workflow of the proposed method.

Figure 1 shows a conceptual workflow of the proposed method. This method is composed of four steps: pre‐processing, artifact splitting, SSIM calculation, and generation of an artifact‐reduced image.

#### 2.B.1 Pre‐processing: denoising

Because this study uses a modified SSIM, which includes only contrast and structure factors neglecting the effect of the luminance factor, the correlations can be sensitive to noise in CT images. As shown in Eqs. (1), (2), (3), (4), this is due to the contrast and structure factors comprising variance and covariance, respectively. Because noise can hinder the identification of regions with less metal artifacts, a denoising process may be necessary. Denoising in this study was performed by applying a simple two‐dimensional Gaussian smoothing kernel.

#### 2.B.2 Artifact splitting

After denoising, an artifact superposition map is synthesized to exclude structural information on the scanned object; only metal artifacts were considered, as mentioned in Section 2.A. This artifact superposition map was constructed by calculating the difference between the two CT images. As shown in Fig. 1(c), the difference between the original and the tilted CT images represents the superposition of metal artifacts from the two images.

#### 2.B.3 Calculation of structural similarity

To select the regions in the two CT images with less metal artifacts, the correlation maps from each reconstructed image and the superposition maps were calculated [Figs. 1(d) and 1(e)]. Correlation values were calculated using the modified SSIM, which includes contrast and structure factors. The patch size to calculate the modified SSIM was set to be 5 × 5 pixels, with the resulting correlation values representing the degree of artifact contamination in the corresponding reconstructed images, which was calculated using Eq. (5). The negative sign in Eq. (5) was utilized to retain the positive correlation of metal artifacts in the tilted CT images, as superposition maps were calculated by subtracting the tilted from the original CT images:

(5) $$\left\{\begin{array}{c} C^{\mathrm{ori}}=SSIM^{\prime }\left(I^{\mathrm{ori}},I^{\mathrm{artifact}}\right)\\ C^{\mathrm{tilt}}=SSIM^{\prime }\left(I^{\mathrm{tilt}},-I^{\mathrm{artifact}}\right) \end{array}\right.,$$

#### 2.B.4 Generation of artifact‐reduced images

As higher values in the correlation map represent greater contamination with metal artifacts in the corresponding reconstructed images, selecting the regions with lower correlation values would lead to relatively more artifact‐free than the two CT images. Therefore, the final artifact‐free image can be generated using Eq. (6):

(6) $$I^{G}=\left\{\begin{array}{cc} I^{\mathrm{ori}}, & if\;C^{\mathrm{ori}}<C^{\mathrm{tilt}}\\ I^{\mathrm{tilt}}, & otherwise. \end{array}\right.$$

### 2.C Experimental conditions

Both simulation and experimental studies were performed to determine the feasibility of the proposed method and to compare its performance with those of existing methods.

#### 2.C.1 Simulation study

**Figure 2:**
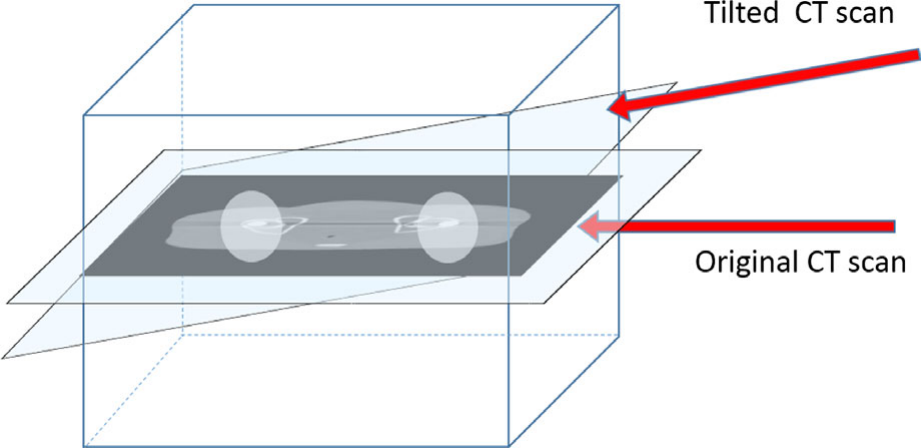
Schematic diagram of an additional tilted CT scan in the simulation study.

**Figure 3:**
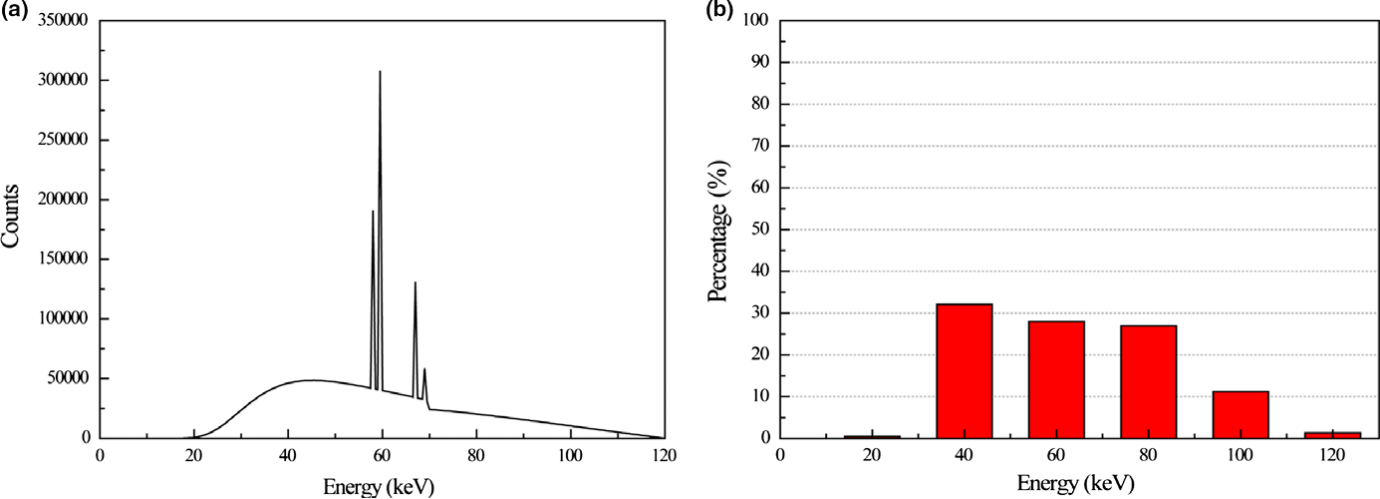
Spectrum of polychromatic x rays in the simulation study. (a) A 120 kVp x‐ray spectrum with tungsten target. (b) Six monochromatic x rays at representative energy bins of 120 kVp.

The simulation study was performed using the pelvic region of an XCAT numerical phantom.^48^ Projection data of the phantom with and without metal implants were generated by circular scanning geometry using Matlab fan‐beam function (The Mathworks Inc., Natick, Massachusetts, USA) under the condition that physical factors of metal artifacts, other than photon starvation, beam hardening, Poisson noise, and Gaussian noise, were neglected. For simulating Poisson noise, random noise from the Poisson distribution function (PDF) with the corresponding pixel values (~10^7^) was added to the intensity data. Gaussian noise was calculated from the Gaussian distribution with the mean as zero and variance as 0.001. Then, the Gaussian noise was added to log‐transformed projection data. To simulate metal artifacts, bilateral metal implants of radius 0.85 cm were inserted into the phantom. Projection data were acquired at gantry tilt angles equal to 0° and 10° to avoid overlapping of metal implants along the beam direction (Fig. 2). The nature of polychromatic x rays was simulated by summing the weighted data of six monochromatic x rays at representative energy bins as shown in Fig. 3(b) (20, 40, 60, 80, 100 and 120 keV, respectively). The x‐ray tube voltage was set at 120 kVp^49^ [Fig. 3(a)]. The distance between the x‐ray source and the isocenter was 400 mm; and the distance between the x‐ray source and the detector was 1100 mm. A total of 720 projection views were obtained over the 360‐degree scanning range.

#### 2.C.2 Experimental study

**Figure 4:**
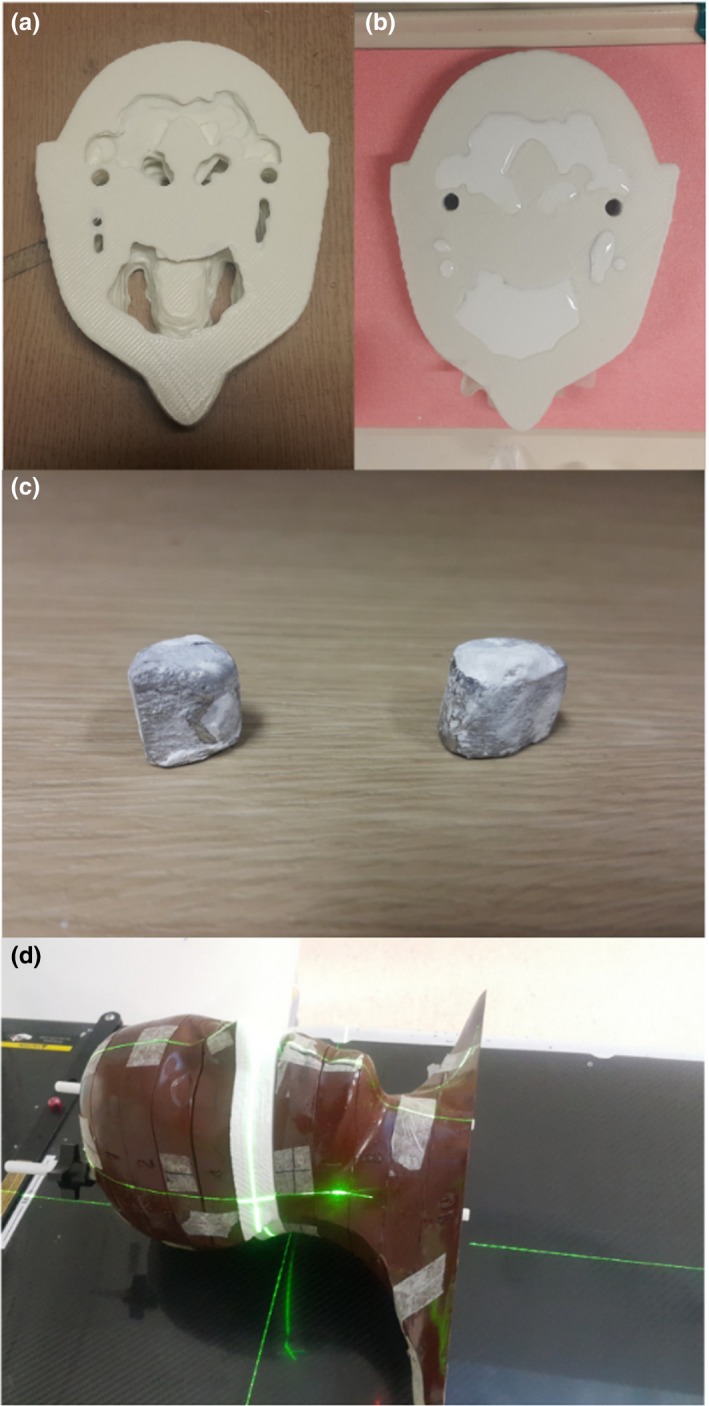
Fabrication of the physical phantom utilized in the experimental study. (a) Slice of a 3D‐printed Rando phantom and (b) after pouring of gypsum. (c) The two metal inserts made of Cerrobend alloy. (d) The assembled Rando phantom.

**Figure 5:**
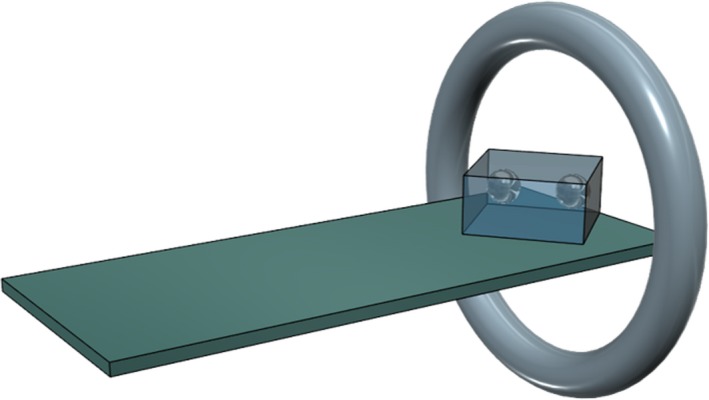
Schematic diagram of scanning geometry for an additional tilted CT scan in the experimental study.

The experimental study utilized the head part of the Rando phantom. Instead of using all the slices of the original Rando phantom, certain slices were replaced by a customized phantom fabricated by the 3D printer [Fig. 4(d)]. This process was performed because the size and location of holes in the original Rando phantom are fixed, making this phantom inappropriate for simulating metal artifacts. Slices containing areas of the nasal and oral cavities were selected and scanned using a 16‐slice CT scanner (GE Lightspeed 16, GE Healthcare, Milwaukee, Wisconsin, USA) to obtain a template of the phantom. CT images were modified to remove bony structures, securing the regions for metal inserts. The resulting surface model was converted to a digital model in the STL format for the 3D printer. The phantom was fabricated by the 3D printer with polylactic acid (PLA) representing all tissues [Fig. 4(a)]. Two metal inserts, each of radius 0.6 cm and made of Cerrobend alloy [Fig. 4(c)], were placed in the gingiva region, and gypsum paste representing the bone was poured [Fig. 4(b)]. Using the resultant phantom, CT scan data were acquired at gantry tilt angles 0° and 15°. However, unlike the simulation study, avoiding metal implants along the beam direction was difficult as gantry tilt is available only in the anterosuperior to posteroinferior direction and in the posterosuperior to anteroinferior direction. Therefore, the phantom was scanned in an oblique direction, being rotated by 15° along the vertical axis as shown in Fig. 5. The x‐ray tube voltage was again set at 120 kVp; the distance between the x‐ray source and the isocenter was 605 mm; and the distance between the x‐ray source and the detector was 1062 mm.

## 3 RESULTS

### 3.A Simulation study

**Figure 6:**
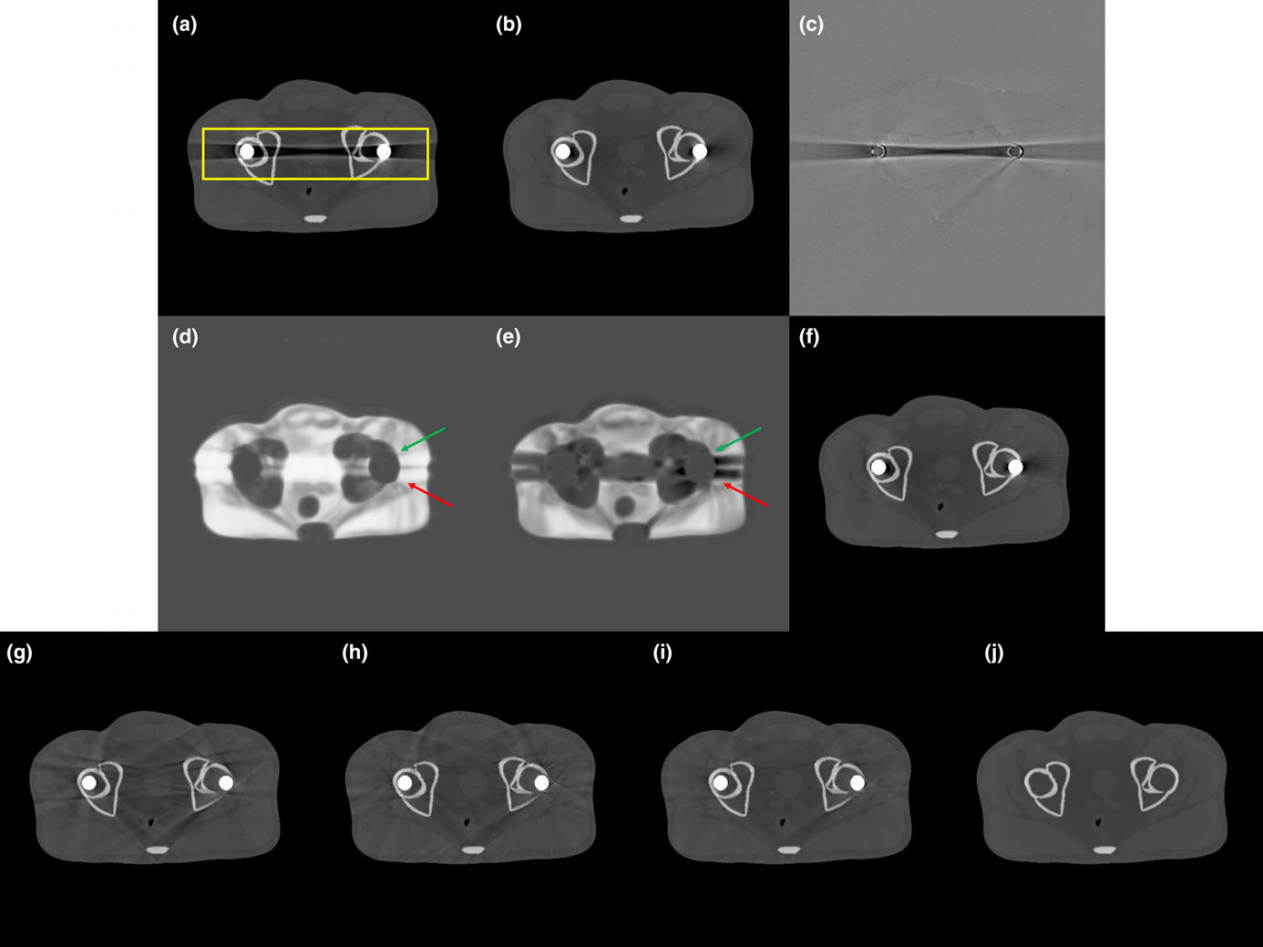
Reconstructed images of the XCAT numerical phantom in the simulation study. (a) and (b) CT images obtained with an (a) ordinary and a (b) tilted CT scan. (c) Difference between (a) and (b). (d) and (e) Correlation maps of (a) and (b), respectively. (f) Image resulting from the proposed method. (g)–(i) Result images obtained by LI‐MAR, NMAR, and RMAR, respectively. (j) Artifact‐free image (reference). The window widths (WW) and window levels (WL) were 0.023 and 0.025 cm^−1^, respectively, for (a), (b), and (f)–(j); 0 and 0.02 cm^−1^, respectively, for (c); and 0.3 and 1.5 cm^−1^, respectively, for (d) and (e).

**Figure 7:**
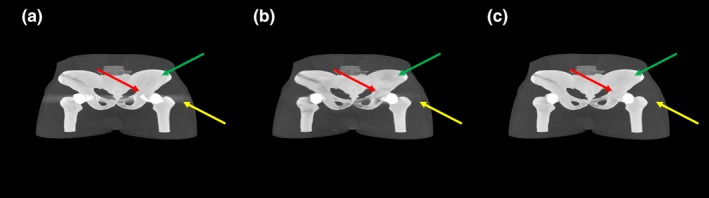
Volume‐rendered 3D images in MIP mode of CT data with an (a) ordinary and (b) tilted CT scan, and a (c) result image from the proposed method.

Figure 6 shows the reconstructed images of the XCAT numerical phantom based on the original and tilted CT scans. Images from the original CT scan showed streaking artifacts caused by the two metallic implants [Fig. 6(a)]. Although artifacts were still visible in the tilted CT scan, they were strongly reduced [Fig. 6(b)], indicating that the tilted CT scans, which avoided the overlap of metal implants along the beam direction, provide complementary information. To select the regions of the two CT images with fewer metal artifacts, we calculated the correlation maps from each reconstructed image and difference map [Figs. 6(c)–6(e)], with Fig. 6(f) showing the image resulting from our proposed method based on the two correlation maps. Two arrows in Fig. 6(d) and f indicate an example of the area where $C^{\mathrm{ori}}$ is smaller than $C^{\mathrm{tilt}}$. The arrows in the volume‐rendered 3D images created by Amira (FEI Visualization Sciences Group, Burlington, Massachusetts, USA) as shown in Fig. 7 reveal that the proposed method successfully selected artifact‐free regions from the two CT images. Specifically as can be seen in Fig. 7, the proposed method effectively removed metal artifacts from the original CT scan (red and yellow) and tilted CT scan (green); notable improvements were achieved for overall regions. For comparison, we implemented three other existing MAR methods: LI‐MAR, normalized MAR^1^, ^21^ (NMAR), and refined MAR (RMAR)^50^, ^51^, ^52^ [Figs. 6(g)–6(i)]. RMAR using optimally‐tuned settings was included via software made available by the authors of this technique. Because the CT system does not provide sinogram data, virtual sinograms were generated from the original reconstructed images and utilized as input to each algorithm. Figure 6(j) shows the reconstructed image with metal artifact‐free data.

Regions‐of‐interest (ROIs) that we used for quantitative evaluation are indicated in Fig. 6. The regions of soft tissue were subjected to the assessments, as determined by mean absolute percentage error (Table 1). The percentage error was 62.98% based on the original CT scan and 9.27% based on the tilted CT scan. By comparison, the percentage errors of LI‐MAR, NMAR, and RMAR were 19.97%, 14.12%, and 11.79%, respectively. Using the proposed method, the percentage error was reduced to 8.52%. The tilted CT scan itself strongly suppressed the metal artifacts as mentioned above; therefore, the proposed method did not show significant improvements compared to the tilted CT scan, in this simulation study. However, considering the overall regions like Fig. 7, these results indicated that our proposed method successfully reduced metal artifacts and outperformed the other methods. The reason why the proposed method yields even a lower error compared to the tilted CT scan is due to the fact that the proposed method recruits regions partly also from the original CT possibly replacing artifacts‐contaminated regions in the tilted CT scan.

**Table 1:** Mean absolute percentage errors in the numerical XCAT phantom

	Mean absolute percentage error (%)	
MAR type	Original	62.98
	Tilted	9.27
	LI‐MAR	19.97
	NMAR	14.12
	RMAR	11.79
	Proposed method	8.52

### 3.B Experimental study

**Figure 8:**
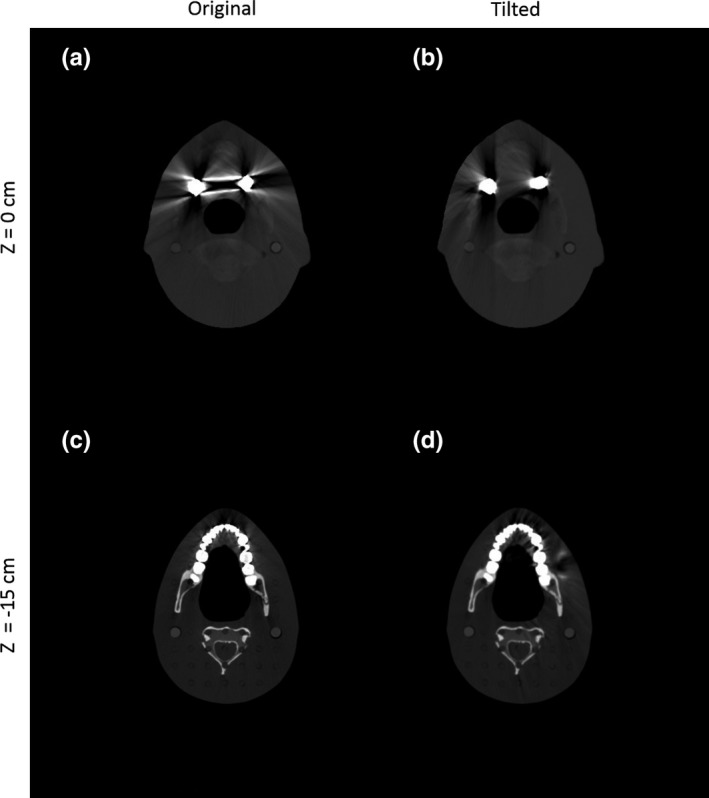
Reconstructed images of the Rando phantom from different slice locations. Top row: CT images obtained with (a) an ordinary scan and (b) a tilted scan at *z* = 0 cm. Bottom row: CT images obtained with (c) an ordinary scan and (d) a tilted scan at *z* = −15 cm.

**Figure 9:**
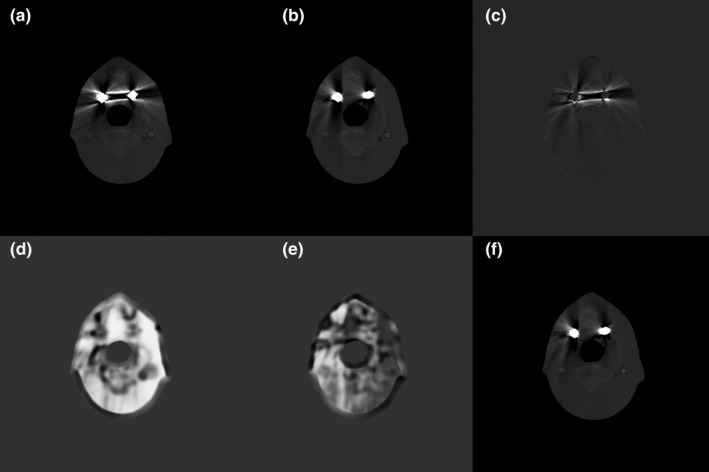
Reconstructed images of the Rando phantom. CT images obtained with (a) an ordinary scan and (b) a tilted scan. (c) Difference between (a) and (b). (d) and (e) Correlation maps of (a) and (b), respectively. (f) Image resulting from the proposed method. The window widths (WW) and window levels (WL) were 850 and 2000 HU, respectively, for (a)–(c) and (f); and 0.3 and 1.5 cm^−1^, respectively, for (d) and (e).

**Figure 10:**
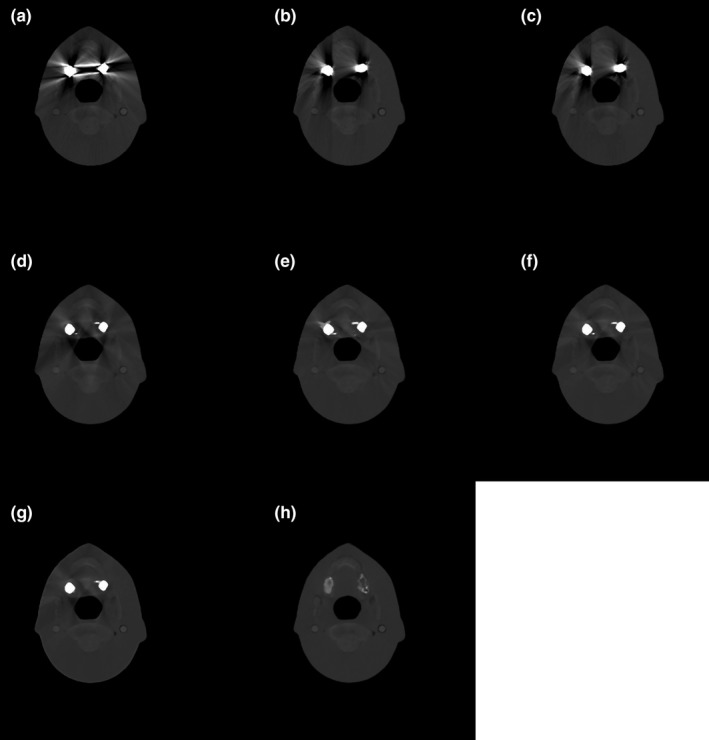
Additional reconstructed images of the Rando phantom. CT images obtained with (a) an ordinary scan and (b) a tilted scan. (c) Image resulting from our proposed method (T‐MAR). (d)–(g) Corrected images obtained by LI‐MAR, NMAR, RMAR, and the modified proposed method (AT‐MAR), respectively. (h) An artifact‐free image (reference). The window widths (WW) and window level (WL) were 850 and 2000 HU, respectively, for (a)–(h).

Figure 8 shows the reconstructed images from different z‐slices of the customized Rando phantom based on the original scan and tilted CT scans. Due to severe metal artifact contamination and inadequate tilt angle relative to the metal implant size, residual metal artifacts in different slice locations were produced from the acquired insufficient complementary information [Fig. 9(b)] as described in Section Methods. Thus, the image resulting from the proposed method contains residual metal artifacts [Fig. 9(c)] despite the success of the algorithm in the simulation study. These issues initiated the application of an additional step in the proposed method. Specifically, the proposed method was combined with an established MAR method, the RMAR method. The proposed workflow was applied once again by employing the intermediate result of the proposed method and the result of RMAR method. Images of the proposed and RMAR methods augmented complementary information are shown in Fig. 10. To distinguish the originally proposed method and the proposed method with the application of an additional step, we named them as tilted CT based MAR (T‐MAR) and augmented tilted CT based MAR (AT‐MAR), respectively. For performance comparison, we also implemented three other existing MAR methods: LI‐MAR, NMAR, and RMAR. Quantitative comparisons of mean absolute percentage error (Table 2) showed that the proposed method (T‐MAR) reduced the percentage error from 94.12% to 46.37%, with an additional step reducing the percentage error to 10.12%. By comparison, the percentage errors of LI‐MAR, NMAR, and RMAR were 45.84%, 34.62%, and 29.93%, respectively. These results showed that the performance of our modified proposed method (AT‐MAR) was superior to those of existing methods.

**Table 2:** Mean absolute percentage errors in the Rando phantom

	Mean absolute percentage error (%)	
MAR type	Original	94.12
	Tilted	60.62
	Proposed method (T‐MAR)	46.37
	LI‐MAR	45.84
	NMAR	34.62
	RMAR	29.93
	Proposed method (AT‐MAR)	10.12

## 4 DISCUSSION AND CONCLUSION

Previous studies showed that metal artifacts cause serious problems in designing RT plans by mitigating tissue visualization in CT images leading to dose calculation anomalies. Tissue differentiation enables medical physicists to accurately determine and contour the target volume and OARs. By doing so, delivering the prescribed dose to the tumor and appropriately sparing the corresponding OARs can be accomplished. This work focused on generating metal artifact‐corrected CT images that can be directly used for creating RT plans. Bilateral hip prostheses and dental implants cases were investigated since dose calculation errors were reported by other literatures to be most significant in these situations. The results of both simulation and experimental studies showed that the proposed MAR method can significantly reduce the metal artifacts by introducing an additional tilted CT scan. Using the modified SSIM as a correlation index, the regions from images of the original and tilted scans, which have less metal artifacts, can be successfully selected to fabricate relatively artifact‐free images. Because the purpose of this study was to demonstrate the feasibility of using an additional tilted CT scan, other cases with numerous metal implants were not included in this work. In all circumstances, the proposed method can effectively prevent the introduction of new artifacts and false structures, which are disadvantages of other existing MAR methods. Although the studies were limited to simple cases, the proposed method outperformed competing methods.

The advantages of the proposed method under conditions of sufficient complementary information include the successful reduction of metal artifacts in the reconstructed images. Under such a condition, unlike the widely used MAR methods that require sinogram data, the proposed method consists of a post‐processing procedure alone, which can be performed without reference to the CT system or reconstruction method. Thus, the calculation time of our proposed method is negligible, although the additional CT scan requires extra time. Under limited conditions, in which the complementary information is insufficient, the proposed method can still reduce metal artifacts by combination with an existing method. Although one of the advantages of the proposed method, the dispensability of sinogram data, is discarded, metal artifacts are still reduced. Additionally, the proposed method may address some of the problems in the existing MAR algorithms. By introducing complementary information, the formation of false structures and new artifacts can be minimized.

The dual energy‐based MAR method^53^, ^54^, ^55^, ^56^ is similar to our proposed method, in that both require double scans. Although a full comparison of the proposed method with the dual energy‐based MAR method is beyond the scope of this work, it should be noted that the dual energy approach is only applicable when the metal artifacts are induced dominantly by beam‐hardening but not by photon‐starvation. The proposed method, in contrast, would be effective in both beam‐hardening dominant and photon‐starvation dominant cases. The proposed method is also compatible with an existing CT equipment, whereas the dual energy‐based method usually requires a particular system specification, for example, two x‐ray tubes producing different voltages or a single x‐ray tube with fast voltage switching.

One drawback of our proposed method is its sensitivity to noise. We demonstrated that denoising in the modified SSIM was successful. Although a simple Gaussian smoothing was sufficient in this study, improved techniques, such as adaptive filtering, would be desirable as these methods can better conserve the structure and edge information of the object. Because complementary information depends on the tilt angle and direction, further studies are needed to optimize the tilt angle and direction in a given clinical situation. Application of the scout image acquired before obtaining the patient's CT image may provide a clue for such optimization.

The major disadvantage of the proposed method is the need for double scans at different tilt angles. The need for two scans would increase the time and dose of radiation exposure. Moreover, extended time allows organ movement to occur, introducing additional artifacts. Fortunately, many regions of metal implantation, such as the head and pelvis, are nearly impervious to the effects of respiration, making the difference in position between two independent scans negligible. Even if this difference is considerable, it is likely to be appropriately dealt with using a post‐processing procedure, such as deformable registration techniques. If the usage of straight deformable registration is inadequate because of critical impact of different appearances of metal artifacts, the bypassing strategy which respectively applying an existing MAR method to two CT data before performing a deformable registration can be another solution.

Although additional radiation exposure due to double scanning can increase the dose of radiation exposure, a more accurate diagnosis may reduce the dose during radiotherapy. Thus, double scanning during diagnostic stage may benefit patients by reducing unnecessary exposure to radiation during the actual treatment. In addition, reducing individual doses while maintaining the total dose may have advantages to patients. Although an additional imaging radiation dose to the patient may constitute a concern, it is thought that low‐dose CT scanning options can be effectively combined with the proposed method without compromising the MAR performance. Even if not, the benefits such as fine target delineation and accurate treatment planning are believed to outweigh the risks related to the increased imaging radiation dose.

Another disadvantage of the proposed method is that its selection of artifact‐free regions and combination of images may introduce non‐continuous features in the resulting images. The noise and intensity of two images will not be exactly the same. Although not likely to have a major effect on the accuracy of the resultant image, the latter may appear artificial. Future studies will include development of a technique to solve this problem, such as smoothing the boundary regions where the two images meet. Further validation of the clinical feasibility and optimization of the proposed method will also require studies on complicated cases with several metal implants and assessment of this method in the patients.

## CONFLICTS OF INTEREST

No conflicts of interest.

## Supporting information


**Data S1.** Metal artifact reduction with an additional tilted CT scan: a preliminary study.

## Figures and Tables

**Figure A1:**
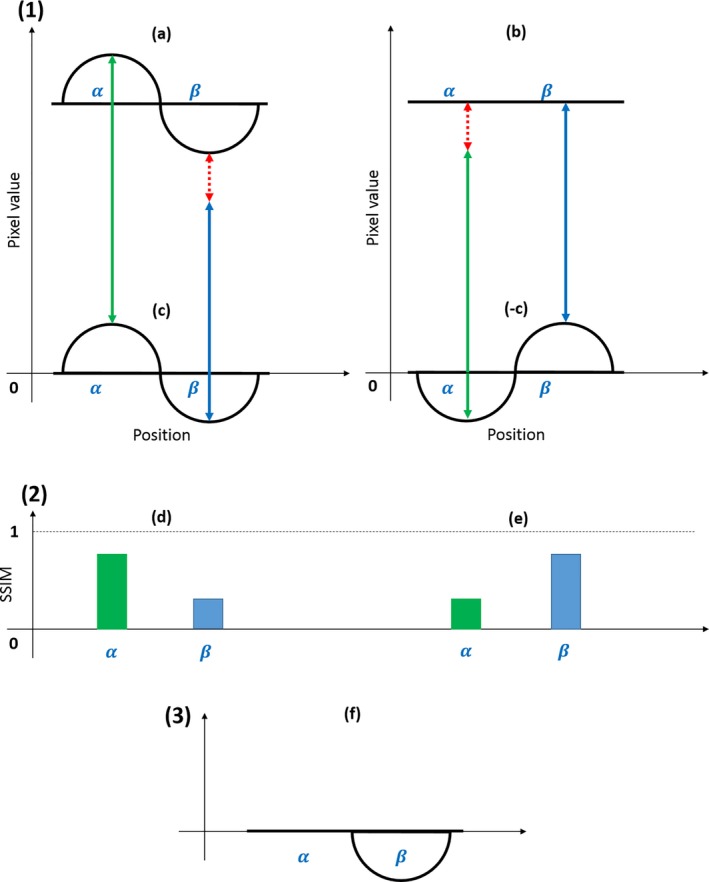
Schematic diagram of calculating correlations with the original SSIM. (1) Line profiles of standard and tilted CT images and their corresponding artifact superposition maps. (2) Line profiles of correlation maps calculated by the original SSIM. (3) Line profile of the resulting image.

**Figure A2:**
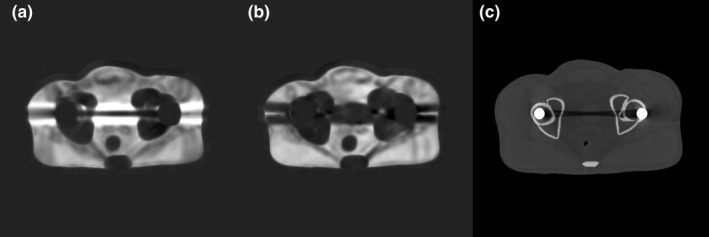
Reconstructed images of XCAT phantom for appendix. Correlation maps of (a) original and (b) tilted CT images. (c) Image resulting from the proposed method using the original SSIM.

## APPENDIX 1

### 1.1

Here, we demonstrate the problem of using original SSIM for calculating correlations between CT images and artifact superposition maps. If metal artifacts, such as white streaks and dark bands, are mainly present in standard CT images but not in tilted CT images, their line profiles can be described as in Figs. A1(1a) and A1(1b). The symbols α and β represent the positions of white streaks and dark bands, respectively, in standard CT images. The line profile of the artifact superposition map, which is the difference between (a) and (b), would appear as in Fig. A1(1c). Calculation of correlations using modified SSIM, which include only contrast and structure factors, would yield resulting values representing only the structural similarities between (a) and (c) and between (b) and (−c). Utilizing the original SSIM, which includes a luminance factor, would yield correlations reflecting the intensities between (a) and (c) and between (b) and (−c). As the difference in intensity decreases, the correlation value increases. At position α, where white streaks are located, the correlation value would be smaller for tilted than for original CT images, because the difference in intensity is smaller for original CT than for tilted images [see green arrows in Figs. A1(1a) and A1(1b)]. At position β, however, the difference in intensity is greater in original than in tilted CT images (see blue arrows), resulting in a smaller correlation value for original than for tilted CT images, despite similarities in contrast and structure. Thus, the dark bands remain in the resultant image [Fig. A2(c)]. These findings indicated that the modified SSIM, without the luminance factor, would be appropriate for calculating correlations in our proposed method.
