# Supplementary material for: An additional tilted‐scan‐based CT metal‐artifact‐reduction method for radiation therapy planning
Source: J Appl Clin Med Phys. 2018 Dec 31;20(1):237–49. doi: 10.1002/acm2.12523 (PMC6333137; doi:10.1002/acm2.12523)
Supplement: Supplementary file 1 — Data S1. Metal artifact reduction with an additional tilted CT scan: a preliminary study. [file ACM2-20-237-s001.pdf]

Metal Artifact Reduction with an Additional Tilted CT Scan: A Preliminary Study

Changhwan Kim [1], Chang Hwan Lee [2], Byung-Chul Cho [2], Chiyoung Jung [2], Seungryong Cho[1], Sang-Wook Lee [2], and Jungwon Kwak [2]

[1] KAIST, Daejeon, South Korea
[2] Asan Medical Center, Seoul, South Korea

INTRODUCTION

- Metal artifacts in CT images degrade the image quality and cause severe errors in dose calculation of radiotherapy planning.
- We proposed a new metal artifact reduction scheme which utilizes an additional tilted CT image.
- We demonstrated feasibility of the proposed method by simulation study with the pelvic part of the XCAT numerical phantom.

METHOD

- The basic idea of proposed method is that metal artifacts in CT images mainly traverse the object along the beam direction in CT scan.
- Therefore, an additional tilted CT scan would provide complementary information where some regions were free of metal artifacts.
- To select the regions where have less metal artifacts, we calculated correlation maps between CT images and their difference map.
- The chosen regions where have lower correlation values would be a template for artifact-free image. Thus, one can generate the best of the information in both two images, resulting in metal artifact-free CT image.
- Instead of original structural similarity index (SSIM), we used a modified SSIM for the correlation map to exclude the effect of luminance measurement.

$$SSIM(x,y) = l(x,y)\,\underbrace{\boxed{c(x,y)\cdot s(x,y)}}_{SSIM'} \quad \text{, where} \quad c(x,y) = \frac{2\sigma_x\sigma_y + C_2}{\sigma_x^2 + \sigma_y^2 + C_2}, \quad s(x,y) = \frac{\sigma_{xy} + C_3}{\sigma_x\sigma_y + C_3}$$

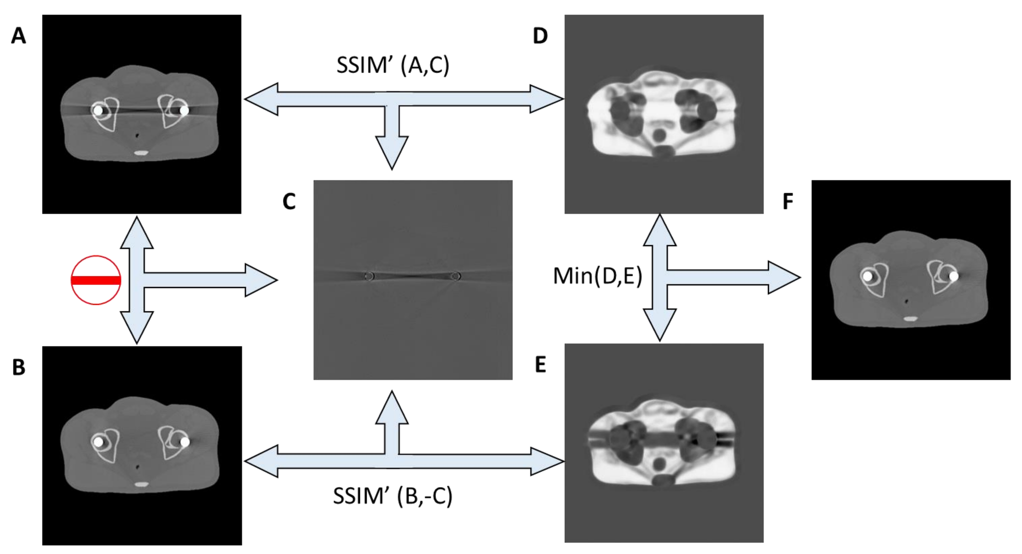

Fig.1. Pipeline of proposed method

RESULTS

- To validate the proposed method, we performed a simulation study with the pelvic part of the XCAT numerical phantom. We added metal artifacts by inserting two metallic implants in the numerical phantom.
- Multislice CT scan data were acquired at 0 and 10 degree (Exp #1), and -5 and 5 degree for the extreme condition that sufficient tilt angle is not guaranteed (Exp #2).
- In case of Exp #2, we introduced an additional step that incorporates with the result of sinogram inpainting to remove residual artifacts caused by insufficient tilt angle.

Exp #1

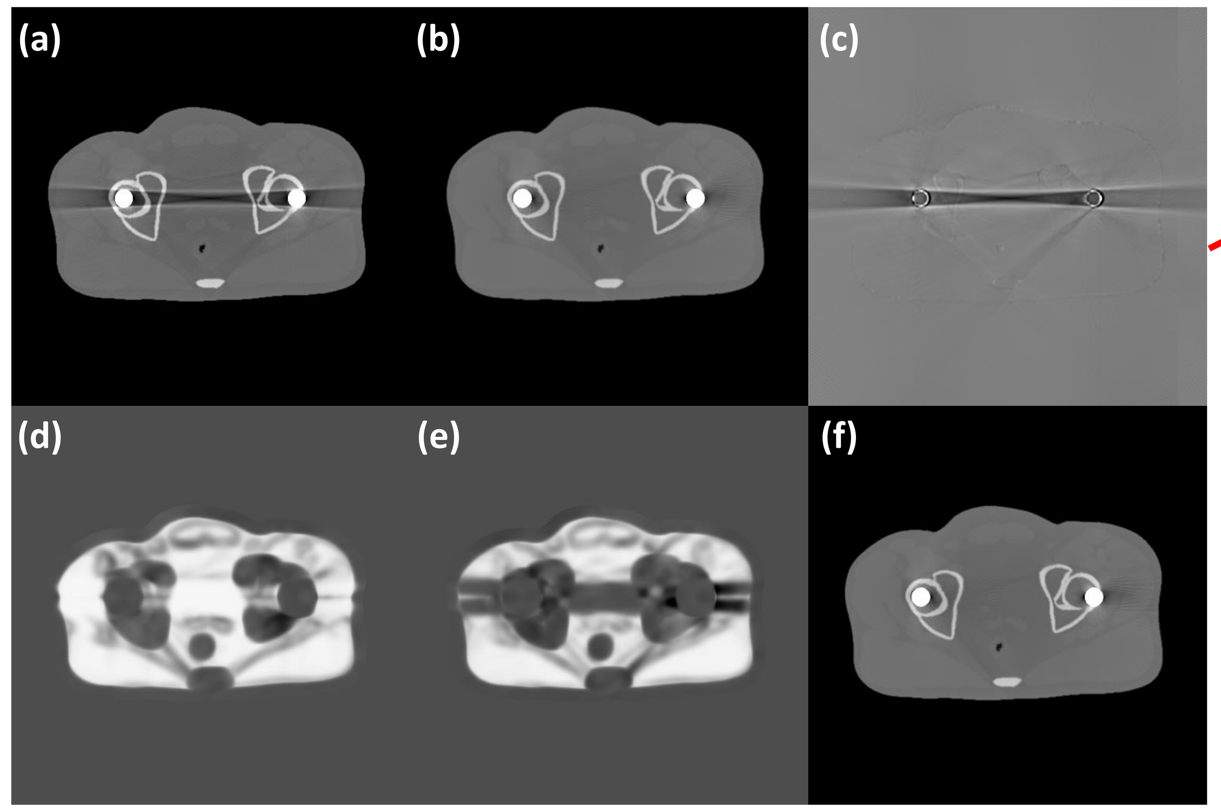

Fig.2. Simulation results of Exp #1. (a) and (b) is the reconstructed images with ordinary scan and tilted scan, respectively. (c) is the difference between (a) and (b). (d) and (e) are the correlation maps of (a) and (b), respectively. (f) is the combined image.

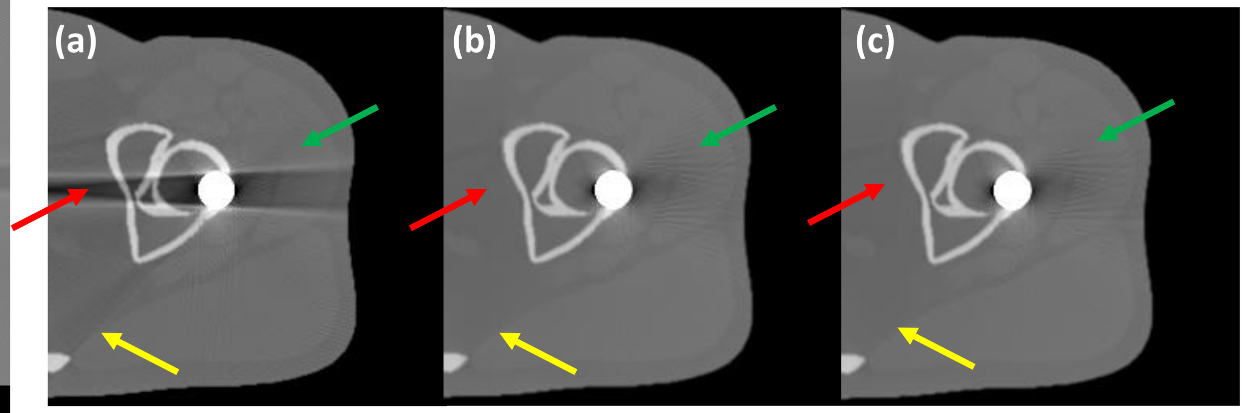

Fig.3. Detailed images of Fig.2.a, Fig.2.b, and Fig.2.f to compare the effect of proposed method.

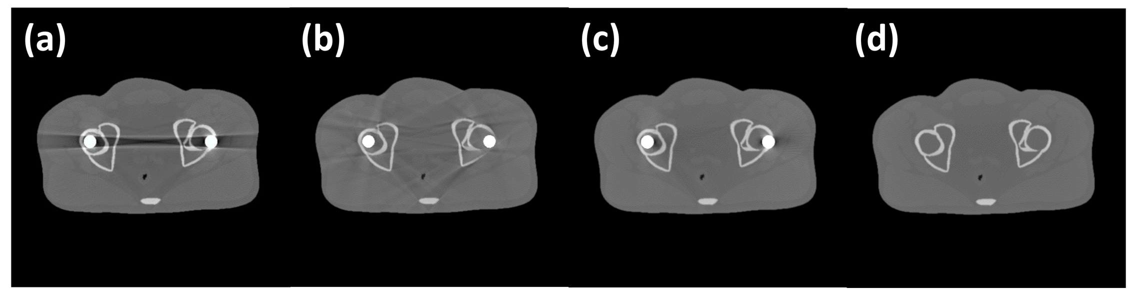

Fig.4. Reconstructed images of XCAT phantom. (a) is the original image, the corrected images are obtained by using (b) sinogram inpainting, and (c) the proposed method. (d) is the artifact-free image. (reference)

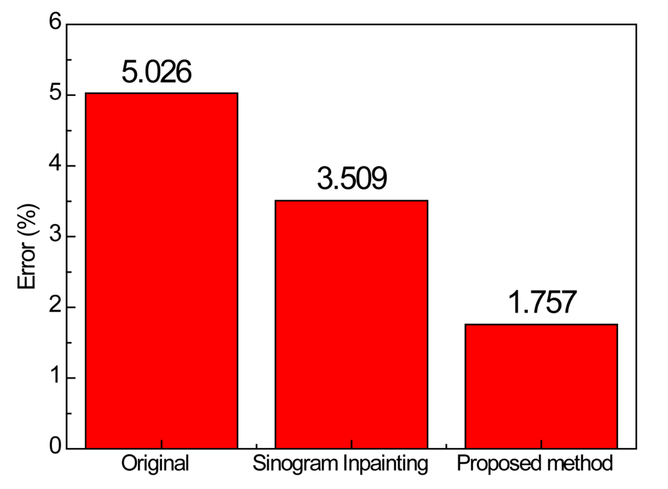

Fig.5. The percentage average error of reconstructed images of XCAT phantom

Exp #2

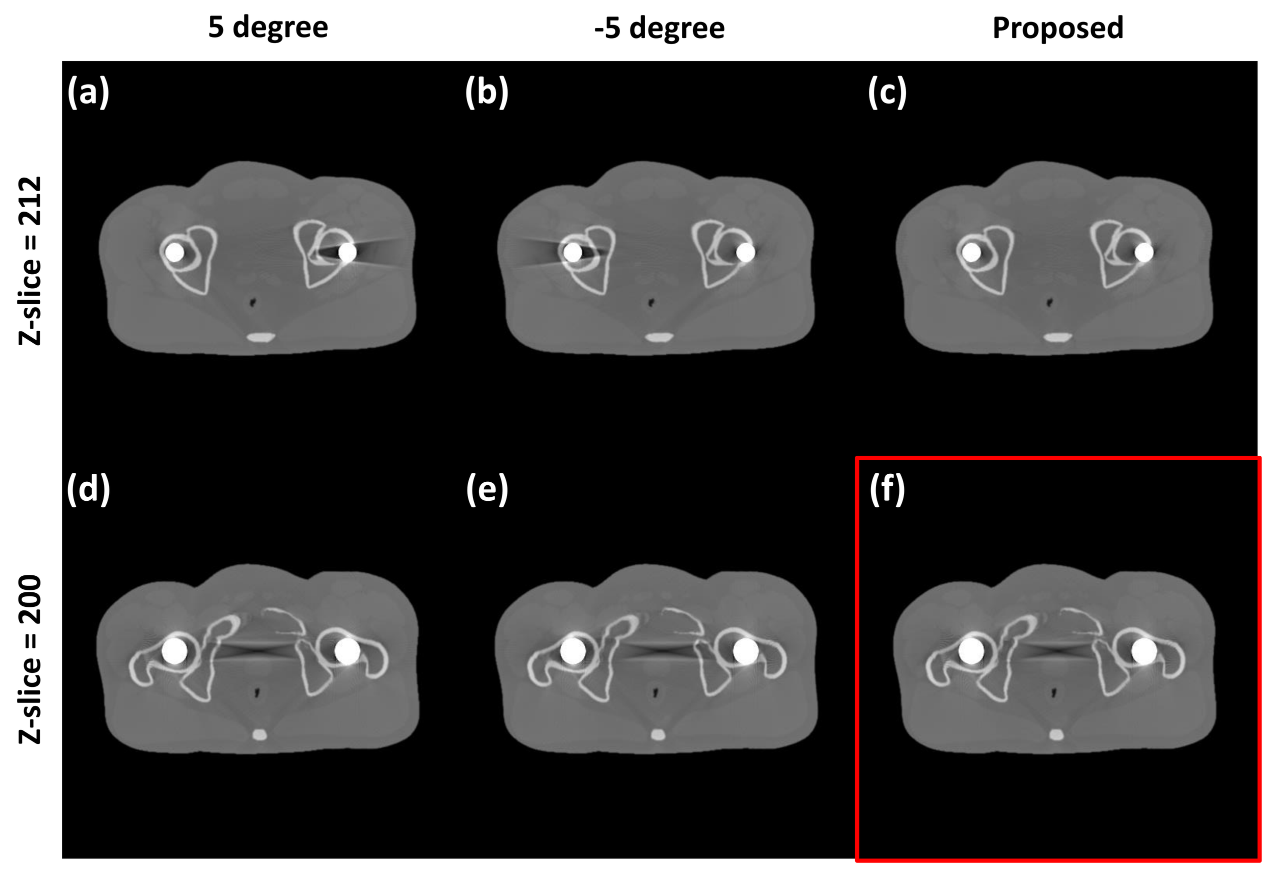

Fig.6. Simulation results of Exp #2. Top row : reconstructed images at z-slice = 212 where proposed method works successfully. Bottom row : reconstructed images at z-slice = 200, where both images have the artifact-contaminated regions due to insufficient tilt angle.

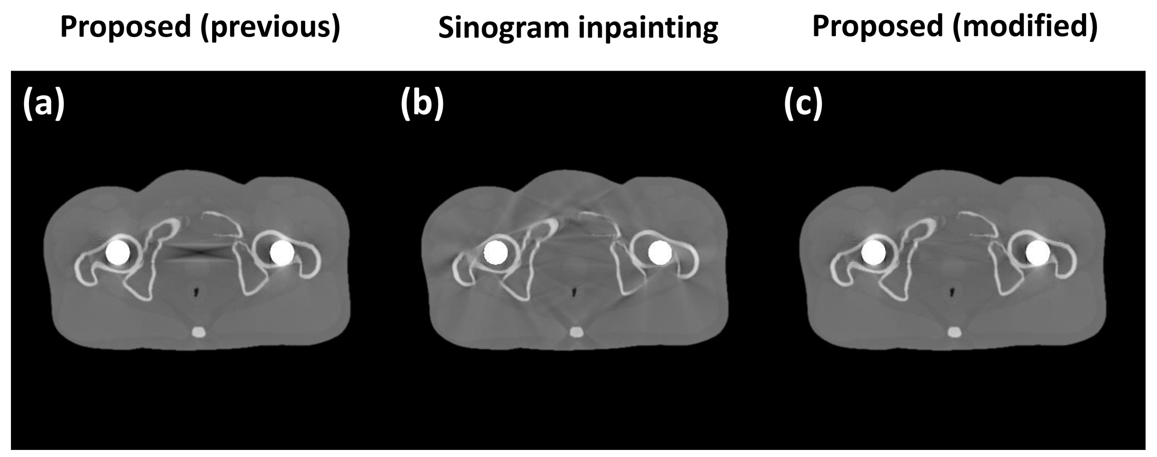

Fig.7. Reconstructed images of XCAT phantom. The corrected images are obtained by using (a) the previous proposed method, (b) sinogram inpainting, and (c) the modified proposed method.

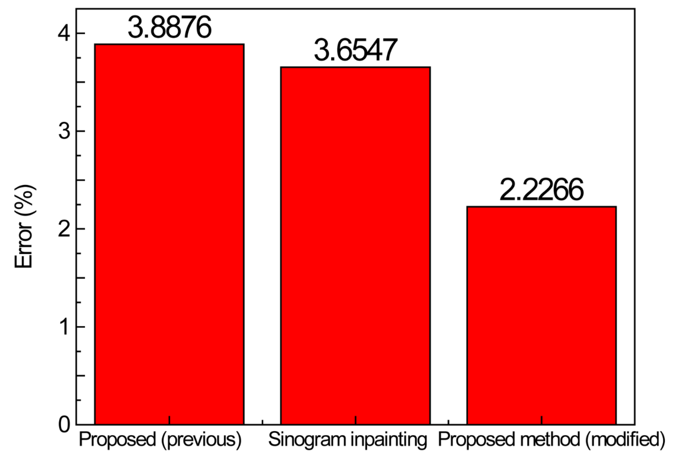

Fig.8. The percentage average error of reconstructed images of XCAT phantom with insufficient tilt angle

CONCLUSIONS

- In this work, we investigated a new metal artifact scheme with an additional tilted CT scan. The results from the simulation study with XCAT numerical phantom demonstrated that proposed method successfully reduces the metal artifacts comparable to the existing methods without burdensome calculations.
- In the ordinary condition where sufficient tilt angle is guaranteed, the proposed method successfully removes the metal artifacts only with reconstructed images in a short time as most other existing methods require sinogram data and burdensome calculations.
- In the harsh condition that both images have artifacts at certain regions due to insufficient tilt angle, we retained the image quality as introducing the result of sinogram inpainting method, although the merit that sinogram data are unnecessary would be discarded.

REFERENCES

1. Wang Z, Bovik A, Sheikh H, Simoncelli E. Image Quality Assessment: From Error Visibility to Structural Similarity. IEEE Transactions on Image Processing. 2004;13(4):600-612.

2. Ballhausen H, Reiner M, Ganswindt U, Belka C, Söhn M. Post-processing sets of tilted CT volumes as a method for metal artifact reduction. Radiation Oncology. 2014;9(1):114.

3. Yanbo Zhang, Xuanqin Mou, Metal artifact reduction based on the combined prior image. arXiv preprint arXiv:1408.5198 (2014).

ACKNOWLEDGEMENTS

This research was supported by the National Research Foundation of Korea (NRF) funded by the Korea government (MSIP) (2014R1A1A2058154 and 2015M2A2A6A02045253).

CONTACT INFORMATION

Changhwan Kim, E-mail : jason514@kaist.ac.kr
